# Early improvement in food cravings are associated with long-term weight loss success in a large clinical sample

**DOI:** 10.1038/ijo.2017.89

**Published:** 2017-05-02

**Authors:** M Dalton, G Finlayson, B Walsh, A E Halseth, C Duarte, J E Blundell

**Affiliations:** 1 School of Psychology, Faculty of Medicine and Health, University of Leeds, Leeds, UK; 2 Orexigen Therapeutics Inc., La Jolla, CA, USA; 3 Cognitive and Behavioural Centre for Research and Intervention, Faculty of Psychology and Educational Sciences, University of Coimbra, Coimbra, Portugal

## Abstract

**Background:**

Food cravings are associated with dysregulated eating behaviour and obesity, and may impede successful weight loss attempts. Gaining control over food craving is therefore a component in the management of obesity. The current paper examined whether early changes in control over food craving (assessed using the Craving Control subscale on the Control of Eating Questionnaire (CoEQ)) was predictive of weight loss in four phase 3 clinical trials investigating a sustained-release combination of naltrexone/bupropion (NB) in obese adults. The underlying component structure of the CoEQ was also examined.

**Method:**

In an integrated analysis of four 56-week phase 3 clinical trials, subjects completed the CoEQ and had their body weight measured at baseline and at weeks 8, 16, 28 and 56. All analyses were conducted on subjects who had complete weight and CoEQ measurements at baseline and week 56, and had completed 56 weeks of NB (*n*=1310) or placebo (*n*=736). A latent growth curve model was used to examine whether early changes in the CoEQ subscales were associated with decreases in weight loss over time. Confirmatory factor analysis (CFA) was used to determine the psychometric properties of the CoEQ.

**Results:**

The factor structure of the CoEQ was consistent with previous findings with a four-factor solution being confirmed: Craving Control, Positive Mood, Craving for Sweet and Craving for Savoury with good internal consistency (Cronbach’s *α*=0.72–0.92). Subjects with the greatest improvement in Craving Control at week 8 exhibited a greater weight loss at week 56.

**Conclusions:**

These findings highlight the importance of the experience of food cravings in the treatment of obesity and support the use of the CoEQ as a psychometric tool for the measurement of food cravings in research and the pharmacological management of obesity.

## Introduction

Food craving is defined as the intense desire to eat a particular food and is thought to be distinct from hunger in that food cravings occur spontaneously, whereas hunger increases in intensity over time spent without food. The experience of food craving is a relatively common phenomenon;^1, 2^ however, the intensity of food craving varies greatly among individuals. More frequent and intense food cravings are associated with loss of control over eating and poor weight management. For example, research has shown that increased food craving is related to higher body mass index (BMI)^3, 4, 5^ and a greater tendency toward binge eating, emotional and external eating.^6, 7, 8, 9^ Therefore, food craving can be seen as existing on a continuum of experience, ranging from mild to extreme,^10^ is present in normal and disordered eating patterns, and may be elicited under a number of conditions.

Research suggests that reducing and managing food cravings is a key component in the management of obesity and successful weight loss maintenance.^11, 12^ For example, in one weight loss intervention trial that targeted overweight/obese individuals with impaired glucose tolerance, greater food cravings at baseline were associated with a higher BMI, more frequent weight loss attempts, greater weight cycling and increased feelings of perceived deprivation whilst dieting.^5, 13^ It is important to note that, in this study, baseline food craving did not predict weight loss success at the end of the intervention. However, the authors did not report the change in the frequency or intensity of food cravings experienced across the intervention. Therefore, whilst food craving and increased desire to eat ‘forbidden’ foods may contribute to poor compliance and adherence with weight loss interventions, early changes in food craving may be a good clinical indicator of weight loss success and the reduction of food craving represents an important aim of antiobesity therapy.

Sustained-release naltrexone/bupropion (NB; Contrave in the United States and Msyimba in the European Union) is indicated as an adjunct to reduced-calorie diets and increased physical activity for weight loss and chronic weight management in obese adults or those who are overweight with at least one obesity-related comorbidity. NB is believed to act via two distinct mechanisms that contribute to weight loss. The first relates to appetite suppression through bupropion-mediated stimulation of pro-opiomelanocortin neurons in the hypothalamus and naltrexone-mediated suppression of the autoinhibitory pathways of the same pro-opiomelanocortin neurons. The second is via the regulation of the mesolimbic dopaminergic pathways to reduce food cravings and enhance the control of eating behaviour.^14, 15^

The primary aim of the current paper was to determine whether early changes in self-reported control over food craving (i.e. Craving Control) was associated with weight loss outcomes by examining those who exhibit early improvements in Craving Control (‘responders’) compared with those who do not exhibit early improvements in Craving Control (‘non-responders’) in a combined analysis of four 56-week phase 3 clinical trials designed to examine the effect of treatment with NB on weight loss in overweight/obese adults. The pharmacological treatment outcomes of these clinical trials have been reviewed and published elsewhere.^14, 16, 17, 18, 19^ Food craving was assessed using the validated Control of Eating Questionnaire (CoEQ). The CoEQ comprises 21 items designed to assess the intensity and type of food cravings an individual experiences.^8^ Recent research has demonstrated that the CoEQ comprises four subscales: Craving Control, Craving for Sweet, Craving for Savoury and Positive Mood;^20^ therefore, a secondary aim of the current paper was to confirm (using confirmatory factor analysis (CFA)) this component structure in an large, independent, treatment-seeking overweight/obese population.

## Materials and methods

Data reported in this paper were pooled from four phase 3 clinical trials conducted for the Contrave Obesity Research (COR) program (see Table 1). Only subjects who completed the trials following treatment with 32 mg naltrexone sustained-release (SR)/360 mg bupropion SR (NB32) or placebo for 56 weeks were included in the current analysis.

### COR program

The COR-I study examined the effect of NB, NB32 (32 mg naltrexone SR/360 mg bupropion SR) and a lower dose of NB16 (16 mg naltrexone SR/360 mg bupropion SR) compared with placebo.^17^ The COR-II study was similar to the COR-I study and differed only in subjects who did not maintain at least 5% weight loss were re-randomised to receive either NB32 or NB48 (48 mg naltrexone SR/360 mg bupropion SR).^16^ The COR-BMOD study examined the effect of combining NB32 or placebo with an intensive behavioural modification program for weight loss.^19^ Finally, the COR diabetes mellitus (COR-DM) study examined the effect of NB32 in patients with type 2 diabetes mellitus.^18^ In each study the drug or placebo was administered daily. All subjects provided written informed consent and the study protocols were approved by each participating institution. Each study complied with Good Clinical Practice standards and the Declaration of Helsinki. Only subjects who completed the trials following treatment with NB32 (32 mg naltrexone SR/360 mg bupropion SR) or placebo for 56 weeks were included in the current analysis.

### Measures

#### Control of Eating Questionnaire

Subjects completed the CoEQ at baseline and at weeks 8, 16, 28 and 56 of the interventions. The CoEQ comprises 21 items for which participants are required to respond according to their experience over the previous seven days. Dalton *et al.*^20^ identified an underlying four-factor structure of the CoEQ with the following subscales: Craving Control (items 9, 10, 11, 12 and 19), Craving for Sweet (items 3, 13, 14 and 15), Craving for Savoury (items 4, 16, 17 and 18) and Positive Mood (items 5, 6, 7 and 8). Four items in the CoEQ are not included in the calculation of the subscales; items 1 and 2 (‘How hungry have you felt?’ and ‘How full have you felt?’, respectively) did not load onto any of the subscales but were retained in the questionnaire to capture sensations of general appetite and items 20 and 21 assess an individual’s perceived level of control over resisting a nominated, craved food item. Twenty items are assessed using 100-mm visual analogue scales and one item (item 20) allows participants to enter their own nominated food.

#### Body weight

Subjects’ body weight was objectively measured using a calibrated scale, with the subject wearing light clothing and no shoes and recorded to the nearest 0.1 kg at baseline and every 4 weeks throughout the 56-week trial period.

### Data analysis

Only subjects who completed the trials following treatment with NB32 or placebo for 56 weeks were included in the current analysis. Those who received NB16 or NB48 or who did not complete treatment with NB32 were not included in the current analysis. All randomised participants with baseline measurements and one or more postbaseline measurements were included in the analysis. Missing data were imputed using the last observation carried forward. The data were tested to ensure that they met the requirements for CFA using the Kaiser–Meyer–Olkin measure of Sampling Adequacy and Bartlett’s test of sphericity. Cronbach’s *α* was calculated to evaluate internal consistency. To examine whether initial changes in Craving Control, Craving for Sweet, Craving for Savoury and Positive Mood (at week 8) were associated with decreases in BMI over time, a Conditioned Latent Growth Curve Model was tested. This technique considers initial levels of the study variable (intercept mean), the intervariability in these levels (intercept variance), the average rate at which participants change (slope mean) and the interindividual variability in that rate (slope variance).^15^ Changes in Craving Control, Craving for Sweet, Craving for Savoury and Positive Mood at week 8 were included in the model as independent variables. To assess the change (slope) in the outcome variable (weight loss), the observations from baseline, week 8, week 16, week 28 and week 56 were used, and hypothesised to decrease over time. Analyses were conducted using the maximum-likelihood estimation method. The following indices were used to assess model fit: *X*^2^; comparative fit index (CFI); Tucker–Lewis index (TLI); root mean square error of approximation (RMSEA), with 90% confidence intervals; and the standardised root mean residual (SRMR).^21, 22^ All analyses reported in the current paper examine the sample as a whole (i.e. NB32 and placebo combined) as the pattern of results was similar across treatment groups. However, the effects were stronger in those treated with NB32. To examine whether early improvements in Craving Control were associated with weight loss over time, subjects were identified as Craving Control responders (those with the greatest Craving Control improvement at week 8) and Craving Control non-responders (those with the lowest Craving Control improvement at week 8) using a tertile split of change in Craving Control response at week 8. An *α*-level of 0.01 was used to determine statistical significance.

## Results

### Sample characteristics

The sample included in the current analysis comprised 2073 subjects (79% female) who were randomly assigned to receive NB32 (*n*=1310) or placebo (*n*=763) and completed the 56 weeks of treatment. There were no differences between the treatment groups with regard to age, sex, race or baseline BMI and weight (see Table 2), allowing the groups to be combined for this analysis.

### Confirmatory factor analysis

Factor analysis was conducted on the CoEQ subjects completed at baseline. Preliminary analysis of the data revealed that all assumptions of CFA were met. There was no evidence of multicollinearity and the Kaiser–Meyer–Olkin measure of Sampling Adequacy (KMO=0.880) and Bartlett’s test of sphericity (*X*^2^(171)=17 530, *P*<0.001) indicated that the sample size and the data were adequate for conducting CFA. CFA was performed using IBM Amos for Windows (IBM, Chicago, IL, USA; v.22). Fit was assessed by the CFI, the TLI and the RMSEA. A model with reasonably good fit can be characterised by values obtained from the following: a CFI >0.90, a TLI >0.90 and an RMSEA <0.08.^23^

A four-factor structure was assumed to exist in line with previous work:^20^ (1) Craving Control, (2) Craving for Sweet, (3) Craving for Savoury and (4) Positive Mood. The first four-factor model, based on Dalton *et al.*,^20^ was satisfactory (*X*^2^ (87)=1503.4, *P*<0.001; CFI=0.92, TLI=0.90, RMSEA=0.076); however, the factor loadings for item 15 (=0.15) on the Craving for Sweet factor and item 16 (=0.26) on the Craving for Savoury factor were too low. Therefore, a second four-factor model was examined with these items removed. The fit of the second four-factor model was good and superior to the first four-factor model (*X*^2^ (87)=952.3, *P*<0.001; CFI=0.95, TLI=0.94, RMSEA=0.069) with each item loading significantly on its respective factor (see Table 3 and Supplementary Figure S1). As removal of items 15 and 16 improved the model, these items were not included in the final Craving for Sweet and Craving for Savoury subscale scores.

Based on the outcome of the CFA, CoEQ subscale scores were calculated as follows: the sum of the items in each subscale was calculated, and divided by the number of items in the subscale to obtain a subscale score. For the Positive Mood subscale, item 6 (‘How anxious have you felt?’’) was reversed. For the Craving Control subscale, the final subscale score was reversed so that a greater score represented a greater level of Craving Control.

### Internal reliability

Regarding internal consistency, the Cronbach’s *α* values for Craving Control, Positive Mood, Craving for Savoury and Craving for Sweet were 0.92, 0.72, 0.78 and 0.85, respectively.

### Early change in CoEQ subscales with weight loss at week 56

An unconditional latent growth model estimating weight loss over time was first conducted. Results indicated a good model fit (*X*^2^_(8)_=589.77, *P*=<0.001; CFI=0.97; TLI=0.97; RMSEA=0.20 (0.19, 0.22), *P*=<0.001; SRMR=0.01). The mean for the intercept factor was estimated to be 32.97 (*P*<0.001). The estimate of the mean for the slope factor was significant (0.37; *P*<0.001), indicating a significant decrease of BMI over time. Moreover, there were significant variance estimates for both the intercept (29.18, *P*<0.001) and slope (0.54, *P*<0.001), which suggested that there was substantial individual variability around both the mean starting point of BMI and the mean rate of BMI change over time. There was a significant estimate for the covariance between BMI intercept and slope (*r*=−0.60), indicating that participants with higher initial BMI tended to present smaller rates of decrease in BMI over time. The conditional latent growth model revealed a good fit (*X*^2^_(20)_=686.5, *P*=0.000; CFI=0.97; TLI=0.95; RMSEA=0.14 (0.13, 0.15), *P*=<0.001; SRMR=0.01). Results indicated that Craving Control was the only subscale that had a significant effect on the initial levels of BMI (*β*=−0.19; *P*<0.001). Regarding the slope of BMI, initial changes in Craving Control were the best significant predictor (*β*=0.17; *P*<0.001); while initial changes in Craving Sweet also had a significant effect on the slope of BMI (*β*=−0.07; *P*=0.043).

### Craving control response and weight loss

**Figure 1:**
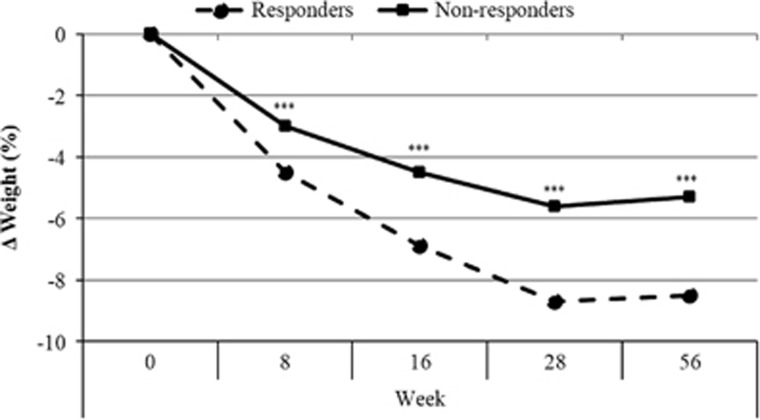
Percentage weight change across the 56-week trial period according to Craving Control response for NB32 and placebo treatment groups combined. ***P<0.001 between craving control responders and craving control non-responders.

Characteristics of the responders and non-responders can be found in Table 4. There were no differences in baseline measures of age (*t* (1257)=0.109, *P*=0.91) or BMI (*t* (1257)=1.46, *P*=0.16). The responders had lower body weight (*t* (1257)=2.85, *P*<0.01) and lower Craving Control scores (*t* (1257)=28.8, *P*<0.001) at baseline compared with the non-responders. Figure 1 shows percentage weight change across the 56-week trial period for responders and non-responders. There was an interaction between time point and group (F (3, 3771)=17.9, *P*<0.001). When this was examined, it was revealed that at each time point responders had a greater percentage weight change compared with non-responders. The same results were found when differences in Craving Control response were examined separately in those treated with NB32 or placebo.

## Discussion

The current paper aimed to determine whether early changes in self-reported control over food craving (i.e. Craving Control) was associated with weight loss outcomes over 56 weeks in a combined analysis of four phase 3 clinical trials that examined the effect of treatment with sustained released combination of NB or placebo on weight loss in obese adults. The latent growth curve model demonstrated that early improvements in Craving Control and reductions in Craving for Sweet throughout the 56-week trial period were predictive of greater reductions in BMI at the end of the trial. When subjects were categorised as responders and non-responders based on their change in Craving Control score at week 8, individuals identified as non-responders (i.e. those who had the lowest Craving Control improvement at week 8) lost ~3–4% less weight compared with individuals identified as responders (i.e. those who had the greatest Craving Control improvement at week 8). This finding is consistent with previous research that has shown food craving and increased desire to eat highly palatable yet restricted foods contribute to poor compliance and adherence with weight loss interventions.^12^ In addition, increased wanting and craving for high-fat sweet foods has been associated with greater binge and disinhibited eating tendencies and a higher level of central adiposity in females with overweight and obesity, indicating that it is a risk factor for weight gain and poor weight loss outcomes.^7, 24, 25, 26^ Promisingly, treatment strategies that target food cravings have proven to be effective in eliciting greater weight loss and preventing weight re-gain. For example, previous research indicates that the use of acceptance-based coping strategies to manage and resist eating in response to food cravings is a characteristic of individuals who successfully maintain their weight loss.^11, 27, 28^ Such strategies may prove to be especially useful for those identified early in an intervention period as experiencing difficulties with eating in response to food cravings.

Taken together, the findings indicate that food cravings have an important role in the treatment of obesity and craving for food presents a target outcome variable for weight loss and prevention of weight gain. Furthermore, the Craving Control subscale of the CoEQ may be useful as an early marker to identify those individuals who may benefit from additional intervention aimed at improving their control over food cravings, which may result in a better weight loss (and control over craving) outcome. Although the contribution of improvements in Craving Control in BMI change may be considered small (explaining 5% of the variance), it is important to note that BMI change is likely to be affected by a large number of individual factors (including genetic, physiological, biological, psychological and social factors) that work not only individually but via interactions to influence the amount of weight lost. In identifying the factors that are important contributors to weight loss success, it is possible to further our understanding of the underlying mechanisms and better support and tailor weight management strategies. Recently, Smithson and Hill demonstrated that resisting eating in response food cravings and reporting greater craving control was predictive of greater weight change over 7 weeks.^21^ Similar to the current study, the amount of variance explained by these factors was 5–7%.

The second aim of the current paper was to determine whether the previously identified component structure of the CoEQ was replicable in a treatment-seeking obese sample. CFA in this clinical population supported a four-factor solution that was coherent with previous work.^20^ There were two items in the current analysis, item 15 ‘How often have you had cravings for fruit or fruit juice?’ on the Craving for Sweet subscale and item 16 ‘How often have you had cravings for dairy foods (cheese, yoghurt)?’ on the Craving for Savoury subscale, that did not load significantly onto their respective factor and were removed from calculation of these subscales. However, these items have been retained in the overall CoEQ scale to allow for the analysis of responses on an item-by-item basis when a specific type of craving is of interest. Therefore, the subscales can be refined, but the four factors identified remained as (1) Craving Control, (2) Craving for Sweet, (3) Craving for Savoury and (4) Positive Mood. The individual subscales had good internal reliability, and the findings demonstrated that the subscales, in particular Craving Control, had predictive validity with regard to weight loss outcomes. These findings support the use of the CoEQ as a psychometric tool for the measurement of food cravings in research and the pharmacological management of obesity.

The primary limitation of the current paper was that while the sample was large, it was limited with regard to the number of male participants and the degree of ethnic diversity. This may restrict the generalisability of the findings. However, the outcomes of the current integrated analysis suggest that the CoEQ is a valid measure of the experience of food cravings, and offers a useful research and clinical contribution to the field in that it samples food craving experiences over a 7-day period, assesses aspects of mood and distinguishes specific, directional cravings from loss of control over eating due to cravings. Food cravings are common experiences but are key in the development of overweight and obesity, weight re-gain and the maintenance of poor eating habits. Treatment and prevention strategies would benefit from identifying individuals who frequently experience intense food cravings and (over)eat in response to them as they may be more susceptible to weight gain and less successful in their attempts to manage their weight. In future research, the CoEQ will provide the means for psychometrically robust outcome measures in clinical trials on obesity and weight management.

## Figures and Tables

**Table 1:** Overview of the COR program

	*COR-I*	*COR-II*	*COR-BMOD*	*COR-DM*
*N* (randomised)	1742	1496	793	505
Population		BMI 30–45 kg m^−2^ or BMI 27–45 kg m^−^^2^ with hypertension and/or dyslipidaemia		BMI 27–45 kg m^−^^2^ with type 2 diabetes
Lifestyle intervention	Standard	Standard	Intensive	Standard
Duration	56 weeks			

Abbreviations: BMI, body mass index; COR, Contrave Obesity Research; COR-BMOD, COR behavioral modification; COR-DM, COR diabetes mellitus; HDL-C, high-density lipoprotein cholesterol; LDL-C, low-density lipoprotein cholesterol. *Note*: Dyslipidaemia, diagnosed with dyslipidaemia, hypercholesterolaemia, hypertriglyceridaemia, hyperlipidaemia, or low HDL and/or classified due to triglycerides ⩾200 mg dl^−1^, LDL-C ⩾160 mg dl^−1^, total cholesterol ⩾240 mg dl^−1^ or HDL-C <40 mg dl^−1^ at baseline; hypertension, diagnosed with hypertension and/or had prescribed antihypertensive medications at baseline.

**Table 2:** Mean (s.d.) subject characteristics for the overall sample

	*NB32 (*n=*1310)*	*Placebo (*n=*736)*
Age (years)	47.0±10.8	47.4±11.1
Sex (% female)	79	79
Race (% white/black/other)	82/14/4	80/16/4
Weight (kg)	101.5±16.9	100.1±15.4
BMI (kg m^−^^2^)	36.2±4.4	36.1±4.2

Abbreviations: BMI, body mass index; NB32, 32 mg naltrexone SR/360 mg bupropion SR; SR, sustained-release.

**Table 3:** Standardised factor loadings for the four-factor CoEQ Model 2

*Item*	*Craving Control*	*Positive Mood*	*Craving for Savoury*	*Craving for Sweet*
Q10. How strong have any food cravings been?	0.88			
Q11. How difficult has it been to resist any food cravings?	0.87			
Q9. During the last 7 days how often have you had food cravings?	0.85			
Q12. How often have you eaten in response to food cravings?	0.83			
Q19. Generally, how difficult has it been to control your eating?	0.75			
Q8. How contented have you felt?		0.86		
Q5. How happy have you felt?		0.73		
Q7. How alert have you felt?		0.66		
Q6. How anxious have you felt?		−0.40		
Q18. How often have you had cravings for savoury foods (fries, crisps, burgers etc.)?			0.94	
Q4. How strong was your desire to eat savoury foods?			0.80	
Q17. How often have you had cravings for starchy foods (bread, pasta)?			0.53	
Q14. How often have you had cravings for other sweet foods (cakes, pastries, biscuits, etc.)?				0.88
Q3. How strong was your desire to eat sweet foods?				0.82
Q13. How often have you had cravings for chocolate and chocolate flavoured foods?				0.73

Abbreviation: CoEQ, Control of Eating Questionnaire.

**Table 4:** Mean (s.d.) subject characteristics for the craving control responders and non-responders

	*Craving control responders (*n=*629)*	*Craving control non-responders (*n=*630)*
Age (years)	46.8 (10.3)	46.8 (10.9)
Sex (% female)	84	75
Race (% white/black/other)	81/16/3	86/13/1
Baseline weight (kg)**	99.5 (15.0)	102.1 (17.2)
Week 56 weight (kg)***	91.1 (16.5)	96.8 (18.7)
Baseline BMI (kg m^−^^2^)	36.0 (4.3)	36.3 (4.4)
Week 56 BMI (kg m^−^^2^)***	33.0 (5.1)	34.4 (5.1)
Baseline Craving Control***	30.6 (13.6)	57.2 (18.8)
Δ Craving Control week 8***	37.9 (12.7)	−7.5 (11.3)

**P<0.01; ***P<0.001.
