# Erratum: Early improvement in food cravings are associated with long-term weight loss success in a large clinical sample

**DOI:** 10.1038/ijo.2017.238

**Published:** 2017-11-09

**Authors:** M Dalton, G Finlayson, B Walsh, A E Halseth, C Duarte, J E Blundell

**Keywords:** Obesity, Weight management

**Correction to:** *International Journal of Obesity* (2017) **41**, 1232–1236; doi:10.1038/ijo.2017.89

Since publication the authors of this paper have requested that it be made open access under the CC BY licence.

