# Supplementary figures and images for: Early improvement in food cravings are associated with long-term weight loss success in a large clinical sample
Source: Int J Obes (Lond). 2017 May 2;41(8):1232–6. doi: 10.1038/ijo.2017.89 (PMC5797930; doi:10.1038/ijo.2017.89)

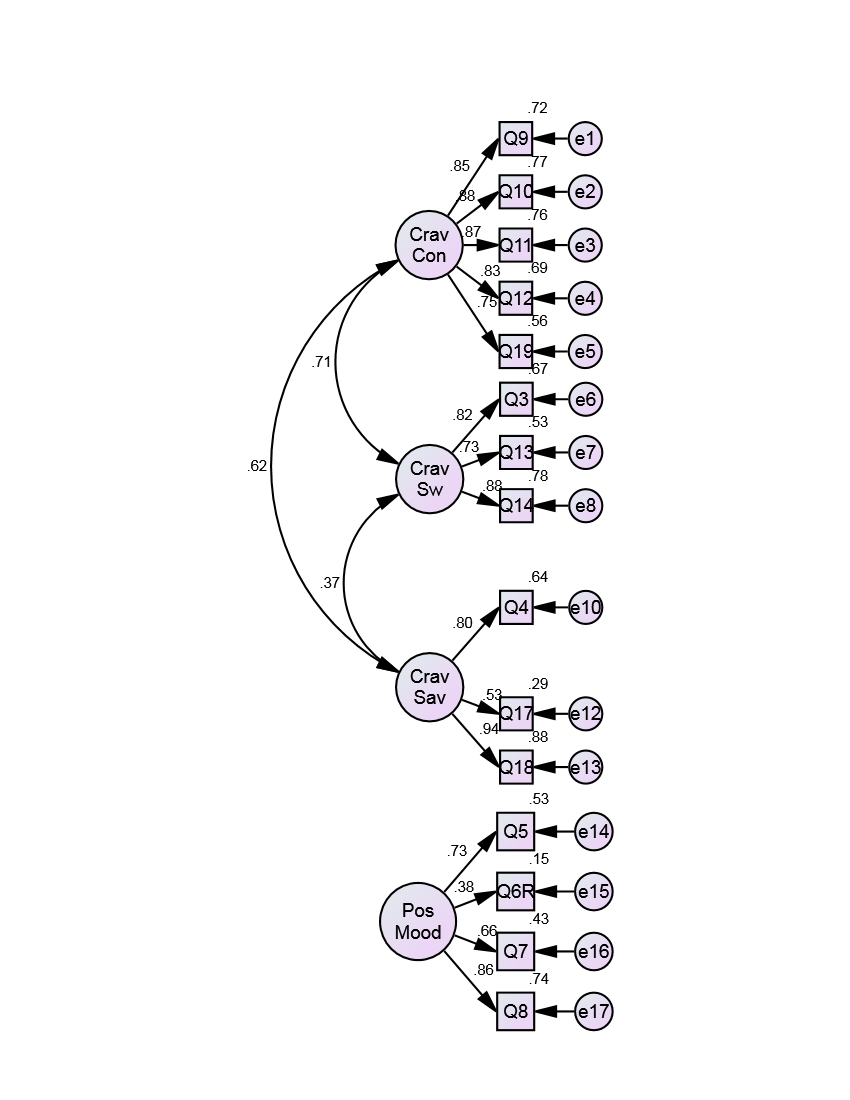


Supplementary Figure 1 Graphical model of confirmatory factor analysis for the CoEQ

Supplement: Supplementary Figure [file ijo201789x1.docx]
